# Supplementary material for: Natural hybridization in heliconiine butterflies: the species boundary as a continuum
Source: BMC Evol Biol. 2007 Feb 23;7:28. doi: 10.1186/1471-2148-7-28 (PMC1821009; doi:10.1186/1471-2148-7-28)
Supplement: Additional File 1 — Hybrids between species of Heliconius and Eueides butterflies: a database. HTML file linking to database of all known wild-caught interspecific hybrid specimens in the Heliconiina, consisting of introductory text, a list of specimens, together with collection data and photographs of the specimens, and links to information about some artificial hybrids and mutants in the group. This is an edited copy of our online database of Heliconius hybrids [102]. To view database, download zip file and extract to a separate folder, then open index.html within that folder. [file 1471-2148-7-28-S1.zip › nummel01.html]

hybrid nummel01


---


Hybrid between *Heliconius numata* ?*superioris* and *H.
melpomene* nr. *meriana*
  
Brazil
  
© K. Brown Jr. (photo MN5)

Return to table of hybrids

To next hybrid
  
To previous hybrid

```
NOTES

No:                      9
Genus of species 1:      Heliconius
Species 1:               numata
Subspecies of species 1: ?superioris
Genus of species 2:      Heliconius
Species 2:               melpomene
Subspecies of species 2: nr. meriana
Sex:                     m
Country:                 Brazil
Locality:                ?Benjamin Constant ?Obidos
Year:
Photo no.:               nummel01
Named hybrid:
Collection:              MN/UFRJ
Collector:               K. Brown photo: MN5
Author/publication:
Notes:                   F1?
```

**Last updated:** 18 October 2003

---
